# Filling up of the Louisville Medical Institute

**Published:** 1837

**Authors:** 


Art. II.—Filling up of the Louisville Medical Institute.

Dr. Cobb, for 13 years a professor in the Medical College of Ohio, has at length (Sept. 13) thought it expedient to resign, and accept the Chair of Anatomy in the Medical Institute now *forming* at Louisville. We congratulate him on the change.

Dr. Locke, still in Europe, has been appointed to the same Chair in the Institute, that he holds in the Medical College of Ohio; and Judge Rowan, the President of the Board of Trustees of the former, has published, that the Doctor's Cincinnati friends are of opinion, that he will accept.

It is honorable to our Medical College, that she should be able to furnish so many professors to neighboring institutions, and even to the East, when they are in a pinch. She is, indeed, a kind of Normal school, for the education of medical teachers, an institution that has long been a *desideratum* in the United States. D.

